# Irrigation of the Large Intestine

**Published:** 1878-01-20

**Authors:** C. W. Dulles


### *IRRIGATION OF THE LARGE INTESTINE.*

By C. W. Dulles, M.D.

Some time since, in reporting a clinical lecture of Dr. Alois Monti, of Vienna (*Philadelphia Medical Times*, August 4, 1877, p. 517), I gave an account of the method of irrigating the large intestine, which he uses so successfully in treating inflammatory conditions of that part of the bowel.

About the time of this publication I met one day Dr. W. C. Barrett, who spoke to me of a severe case of infantile enteritis then under his care. The child was aged six months; had been very ill, vomiting and purging incessantly; had at that time a rapid, feeble pulse; was in a state of extreme depression, and he thought must die. He had employed the usual remedies—chalk, bismuth, opium, aromatic and astringent tinctures, with enemata of laudanum and acetate of lead; but all apparently in vain. Consequently he was quite ready, at my suggestion, to try the plan of irrigating the large intestine alluded to. This he carried out with a so-called fountain syringe, to the tube of which was attached a flexible male catheter. This being introduced into the rectum, cool—not cold—water was allowed to flow from a height of about two feet until good distention was secured, when with scarcely any pres sure the end of the catheter passed smoothly through and beyond the sigmoid flexure, going well up into the descending colon. The water was now allowed to flow steadily on until about a pint and a half had entered, and inspection and percussion showed a large portion of the colon to have been filled. All medicine was then stopped, except mistura cretæ, and for food milk was allowed, and the sucking of a piece of slightly-roasted beef.

The next day there was a marked improvement. "The method," says Dr. Barrett, worked like a charm." He now repeated the irrigation, using not quite so much water. The improvement continued, and convalescence was so rapid that he considered the child well in six days. The vomiting and purging were gone, it was able to suck and digest properly, and the only treatment used afterwards was a short tonic course to assist nature in repairing the inroads made by the disease upon the child's general condition.

The success achieved in this case seemed so plainly dependent upon the procedure desciibed that it furnishes me a welcome opportunity to recommend it to others more explicitly than in the previous article. I have seen it used by Monti in a variety of disorders of the large intestine, as well as for the expulsion of worms and flatus, and always with good results.

In inflammatory conditions of the colon in children he has used solutions of nitrate of silver, such as have been recently recommended in the treatment of dysentery in adults by Prof. H. C. Wood, (see *Philadelphia Medical Times*, October 27, 1877) but decidedly prefers less powerful astringents when—which is very rarely—any are required. In such a case he is apt to select alum in a one or two per cent. solution, sometimes adding a few drops of laudanum. In general, however, he confines himself to the use of simple water, beginning with a temperature barely cool and descending with the successive irrigations till it is about that of spring-water.

He never uses a predetermined quantity, but allows enough to flow in to fill the whole colon "*to the valvula coli.*" In children not yet weaned he finds more than two pints may be used; older children require up to twice this quantity. The quantities used in Prof. Wood's cases of adults were very small compared to these.

It should be stated, in regard to the mode of effecting irrigation, that Monti strenuously opposes the use of a syringe of any kind. The intermittent and uncertain action of these provokes resistance on the part of the intestine, and he thinks may do harm. The securing of an even and easily-regulated hydrotatic pressure is an essential feature of his method. Still more essential is the distention of the rectum with fluid before attempting to pass the tube through the sigmoid flexure. This precaution secures the smoothing out of the folds of mucous membrane and straightens the curves of the flexure, thus rendering the passage of the tube perfectly safe and easy.

Whatever variety of opinion there may be in regard to the possibility of sending an injection beyond the sigmoid flexure, there can be none in regard to the feasibility of "irrigation" by any who have tried or seen it.

Finally, I may say that no special position of the patient is necessary, though it is well, if convenient, to have the pelvis a little elevated. The steps of the procedure are sufficiently indicated in the case given above.

---

TANNIN AS A DEODORIZER OF IODOFORM.—Dr. J. R. Cole writes to *New Remedies:* "Having accidentally discovered that tannin will deodorize iodoform, I take pleasure in making known this fact to you, and through you to the profession. I use it in equal parts, as an application to chancroids, and to old, offensive ulcers.

